# A Comprehensive and Improved Definition for Hospital-Acquired Pressure Injury Classification Based on Electronic Health Records: Comparative Study

**DOI:** 10.2196/40672

**Published:** 2023-02-23

**Authors:** Mani Sotoodeh, Wenhui Zhang, Roy L Simpson, Vicki Stover Hertzberg, Joyce C Ho

**Affiliations:** 1 Public Health Research Institute of University of Montreal University of Montreal Montreal, QC Canada; 2 School of Nursing Emory University Atlanta, GA United States; 3 Department of Computer Science Emory University Atlanta, GA United States

**Keywords:** pressure ulcer, decubitus ulcer, electronic medical records, bedsore, nursing, data mining, electronic health record, EHR, nursing assessment, pressure ulcer care, pressure ulcer prevention, EHR data, EHR systems, nursing quality

## Abstract

**Background:**

Patients develop pressure injuries (PIs) in the hospital owing to low mobility, exposure to localized pressure, circulatory conditions, and other predisposing factors. Over 2.5 million Americans develop PIs annually. The Center for Medicare and Medicaid considers hospital-acquired PIs (HAPIs) as the most frequent preventable event, and they are the second most common claim in lawsuits. With the growing use of electronic health records (EHRs) in hospitals, an opportunity exists to build machine learning models to identify and predict HAPI rather than relying on occasional manual assessments by human experts. However, accurate computational models rely on high-quality HAPI data labels. Unfortunately, the different data sources within EHRs can provide conflicting information on HAPI occurrence in the same patient. Furthermore, the existing definitions of HAPI disagree with each other, even within the same patient population. The inconsistent criteria make it impossible to benchmark machine learning methods to predict HAPI.

**Objective:**

The objective of this project was threefold. We aimed to identify discrepancies in HAPI sources within EHRs, to develop a comprehensive definition for HAPI classification using data from all EHR sources, and to illustrate the importance of an improved HAPI definition.

**Methods:**

We assessed the congruence among HAPI occurrences documented in clinical notes, diagnosis codes, procedure codes, and chart events from the Medical Information Mart for Intensive Care III database. We analyzed the criteria used for the 3 existing HAPI definitions and their adherence to the regulatory guidelines. We proposed the Emory HAPI (*EHAPI*), which is an improved and more comprehensive HAPI definition. We then evaluated the importance of the labels in training a HAPI classification model using tree-based and sequential neural network classifiers.

**Results:**

We illustrate the complexity of defining HAPI, with <13% of hospital stays having at least 3 PI indications documented across 4 data sources. Although chart events were the most common indicator, it was the only PI documentation for >49% of the stays. We demonstrate a lack of congruence across existing HAPI definitions and *EHAPI*, with only 219 stays having a consensus positive label. Our analysis highlights the importance of our improved HAPI definition, with classifiers trained using our labels outperforming others on a small manually labeled set from nurse annotators and a consensus set in which all definitions agreed on the label.

**Conclusions:**

Standardized HAPI definitions are important for accurately assessing HAPI nursing quality metric and determining HAPI incidence for preventive measures. We demonstrate the complexity of defining an occurrence of HAPI, given the conflicting and incomplete EHR data. Our *EHAPI* definition has favorable properties, making it a suitable candidate for HAPI classification tasks.

## Introduction

### Background and Significance

#### Hospital-Acquired Pressure Injury, a Key Nursing Metric

Localized damage to the skin or underlying tissues characterizes pressure injury (PI). PI is typically found over a bony prominence or under a medical device and can be caused by lying down or sitting in one place for too long without much movement [1,2]. Hospital-acquired PI (HAPI) is classified according to the PI stage and the time of its development or progression. HAPI is associated with extended hospital stays, high readmission rates, reduced quality of life, and mortality [3]. HAPI is the most frequent preventable adverse event in hospitals according to the Center for Medicare and Medicaid (CMS) and the second most common claim in wrongful death lawsuits [3]. CMS and the Agency for Healthcare Research and Quality (AHRQ) consider HAPI a “never event,” that is, events with profound financial penalties to providers on reimbursement [4]. More than 2000 US hospitals are part of the National Database of Nursing Quality Indicators program to measure nursing quality metrics, that is, events that are directly associated with the quality of nursing care. The National Database of Nursing Quality Indicators requires participating facilities to perform a quarterly survey of patients to estimate the incidence of HAPI [2]. Thus, accurate information on the incidence of HAPI in a health care unit is critical for assessing nursing quality and planning by hospital administrators.

#### Electronic Health Records and HAPI Identification, Opportunities, and Challenges

Electronic health records (EHRs) provide extensive information on existing and new PIs, including diagnosis codes; characteristics in structured charts, such as stage, depth, and location of PIs; and PI keywords in semistructured or unstructured clinical notes. Automatic detection of HAPI in EHRs using computational models facilitates clinical decision-making and patient care [5]. Predictive models for HAPI depend on the quality, dependability, and consistency of the data set. However, the complexity and subjectivity of PI screening, detection, and staging impact the reliability of PI documentation. PI documentation reliability also depends on the competency and continuity of nursing staff and their roles as well as changes in data entry or the EHR system.

Despite advances in prevention and treatment, HAPI persists and is difficult to identify from EHRs. Data sources provide contradictory information on PIs. Furthermore, the predictive model accuracy relies heavily on the definition of HAPI and accurate labels. Previous studies using EHR data have used inconsistent definitions of the HAPI. Some describe medical conditions that indicate HAPI [2,6,7]; some identify HAPI in all records associated with a hospital stay [7-9]; and others use prior laboratory data to predict HAPI [10].

Inconsistent HAPI labels adversely impact the model performance in HAPI classification and complicate comparison of multiple models. Thus, the correct identification of HAPI labels from EHR data is essential for HAPI studies. Assessing the performance of machine learning models for HAPI tasks using fixed benchmark data requires accessing appropriate clinical data from EHR databases, unifying multiple data sources, and using them consistently with regulatory guidelines. Here, we propose a HAPI definition that meets these requirements.

### Toward a Unified HAPI Definition and More Accurate HAPI Classification

We illustrate the challenges in detecting HAPI in EHRs using the Medical Information Mart for Intensive Care III (MIMIC-III) [11] as a case study. MIMIC-III is one of the most widely used open benchmark data sets, built over CareVue and Metavision EHR systems that encompass approximately 59,000 hospital stays. The patient data include demographics, vital signs, laboratory results, physiological measurements, diagnoses, and clinical and nursing notes.

This study highlights the gaps between existing HAPI definitions for MIMIC-III and the guidelines set by CMS and other regulatory bodies. We propose the Emory HAPI (*EHAPI*) definition, which better adheres to the regulatory guidelines. We then demonstrate the impact of our improved definition in training a more accurate HAPI classification model. The classification performance was evaluated using a manually labeled set from nurse annotators as a proxy for the HAPI ground truth.

Our main contributions are as follows:

1. An improved HAPI definition that leverages diverse data sources and accounts for their reliability while adhering more closely to clinical guidelines
2. Illustrating the impact of the noncomprehensive HAPI definition on training a HAPI prediction model

To achieve these objectives, we pursue the following steps: (1) describe challenges in finding evidence for HAPI within different sources in the MIMIC-III data set, (2) use nursing expertise with clinical information to prioritize and combine conflicting data sources, (3) establish core parameters for a practically reasonable HAPI definition, and (4) determine the impact of the definition on the performance of tree-based and neural network–based HAPI classifiers.

## Methods

### Overview of Data Sources for PI in Hospital Stays

There are 4 major sources of EHR data that may contain information on PI: patient chart events, diagnosis codes, notes, and procedures performed.

#### Chart Events

Chart events constitute the largest portion of structured clinical data and include many medical services, including laboratory tests, vital signs, nurses’ assessments, and general indicators such as patient mental status. Chart events are time-stamped and provide information on the time and order of events during the hospital stay.

#### Diagnosis Codes

For billing purposes, each hospital stay contains a limited set of diagnosis codes. These codes usually include the most important diagnoses during the hospital stay; however, financial concerns and imperfect mapping of clinical findings to predetermined codes can impact this.

#### Notes

Clinical notes include any unstructured text information such as radiography reports, electrocardiogram reports, discharge summaries, admission notes, and daily notes made by the care team.

#### Procedure Codes

Procedure codes indicate timed medical services and surgeries.

PI staging is a core element of the HAPI deterioration status from admission to discharge. Most modern clinical information systems, including the Metavision and CareVue clinical information systems in the MIMIC-III and Emory Healthcare’s clinical data warehouse, contain PI staging events and notes. Multimedia Appendix 1 [12] summarizes the details of the PI data sources in the MIMIC-III.

### Ideal HAPI Criteria Based on Guidelines

Regulatory authorities identify HAPI using many elements, including the presence of PIs at admission and discharge, changes in stages, unit transfers during admission, and patient death. CMS provides several inclusion and exclusion criteria for HAPI [13-15]. One inclusion criterion is the presence of one or more new or worsened PIs at discharge compared with admission. This includes stages 2 to 4, or PIs not staged owing to slough or eschar, nonremovable dressing or device, or deep tissue injury. Another inclusion criterion is an unstageable PI on admission that is later staged. This is coded on discharge assessment as “present on admission,” with the earliest assessed numerical stage. A patient stay is excluded if data on new or worsened stages 2, 3, and 4, or unstageable pressure ulcers, including deep tissue injuries, are missing on the planned or unplanned discharge assessment. In addition, a patient stay is excluded when the patient died during the hospital stay.

The standard practice for newly admitted patients is the completion of admission assessment, as close as possible to the time of admission and within 24 hours. AHRQ also suggests “performance of comprehensive skin assessment within 24 hours of admission” to accurately assess PI rates [16]. The National Pressure Injury Advisory Panel (NPIAP) reference guide [17] defines the facility-acquired rate as the “percentage of individuals who did not have a pressure injury on admission who acquire a pressure injury during their stay in the facility.”

### Existing MIMIC-III HAPI Case Definitions and Their Limitations

There are 4 existing HAPI definitions for MIMIC-III, which are summarized in the subsequent section. Detailed flowcharts for the various definitions are provided in Multimedia Appendix 1.

Recurrent additive network for temporal risk prediction (CANTRIP) [10] focused on predicting HAPI 48 to 96 hours before its first appearance, or date of event (DOE). The DOE was defined as the first occurrence of either mention of PI-related keywords in time-stamped hospital notes or a PI staging chart event (≥stage 1) >48 hours after admission. Other stays without a DOE were marked as controls. Unfortunately, the CANTRIP case definition included deceased patients and healed or improved PIs.

Cramer et al [6] sought to develop a screening tool for PI by using the first 24 hours of data. They identified HAPI cases using only the PI staging chart events occurring 24 hours after admission. It excluded stage 1 PIs and “unable to stage” and deep tissue injury PIs. Similar to CANTRIP, the Cramer case definition included deceased patients and healed or improved PIs. Other stays constituted the control group.

Sotoodeh et al [9] explored the use of negation preprocessing on clinical text to detect PI. Case patients were defined using International Classification of Diseases (ICD)-9 codes or PI-specific keywords in the clinical notes. Similar to CANTRIP and Cramer definitions, deceased, healed, or improved PIs were included in the case definition. However, in contrast to the CANTRIP and Cramer definitions, they did not consider PI staging chart events. Control stays were defined as the absence of both ICD-9 codes and PI-specific keywords.

Cox et al [7] focused on identifying appropriate risk factors for PI by using selected variables from the existing literature. They identified a subset of patients who did not have preexisting PI on admission. However, the inclusion and exclusion criteria for identifying HAPI were not explicitly mentioned and are therefore not presented here.

Other studies have focused on predicting HAPI by using other EHR databases. Ranzani et al [8] focused on predicting PI within 30 days of intensive care unit admission in the first 24 hours. They excluded patients who had a preexisting PI on admission or developed PI within the first 48 hours. The case definition was similar to that of CANTRIP, except that notes were not used. Song et al [18] also proposed an early assessment tool for PI risk using 28 relevant features from existing literature. However, the case definition was not discussed in detail. Finally, Hyun et al [19] developed a machine learning model to predict the HAPI. HAPI cases were defined as those containing an ICD-9 code associated with a PI.

### EHAPI Case Definition in MIMIC-III

**Figure 1:**
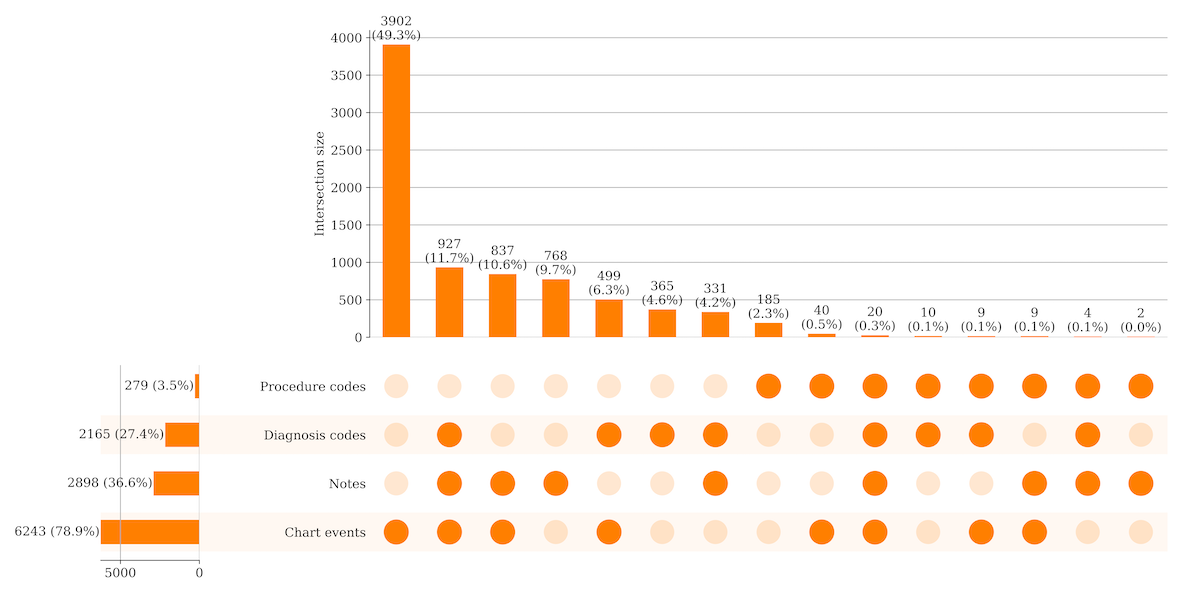
Medical Information Mart for Intensive Care III (MIMIC-III) data sources consistency for pressure injury (PI) hospital stays.

On the basis of existing and ideal HAPI criteria, we identified several essential elements to create a HAPI case definition using EHR data and applied it to MIMIC-III. MIMIC-III has limitations, that is, incongruence of data sources regarding PIs presence and complexity of extracting stage data to verify PI deterioration criteria from admission to discharge, for stays with only comments about PIs in nursing notes and not as timed structured data (Figure 1 and section *Limitations and Future Work*). These limitations inform this exemplar MIMIC-III HAPI case definition.

For the HAPI criteria, we can include or exclude deceased patients, set a minimum age, and consider either 24 or 48 hours from admission to determine admission PIs status. Further decisions for HAPI criteria include the set of clinical events related to PI staging data, the minimum numerical stage for HAPI, numerical stage values assigned to deep tissue injury and unstageable PIs, and the inclusion or exclusion of healed or improved PIs at discharge. Moreover, in addition to staging events, to determine HAPI labels, we considered the presence of certain keywords or diagnosis codes in the notes. We propose a more comprehensive version of the previous definitions, *EHAPI.* The *EHAPI* definition is based on the updated version of the HAPI criteria as determined by the CMS, NPIAP, and AHRQ guidelines [2,14-16]. We extracted data from the admission, patient, and intensive care unit stay tables in the MIMIC-III to construct features and remove irrelevant stays from our analysis.

Our case definition considered only new PIs or PIs that deteriorated by discharge, which required determining the PI stages at admission and discharge for each hospital stay. If a patient had multiple hospital stays, we treated each stay separately. Moreover, in MIMIC-III, a hospital stay encompasses ≥1 intensive care unit stays. Staging occurred within 24 hours of admission. Stage 4 is deep PI, and “unable to stage” was coded as 0. In the absence of the PI stage information at admission, the stage was set to 0. Discharge stage was set as the last recorded stage above 2 occurring later than 24 hours of admission, considering deep tissue injury as stage 3 and “unable to stage” as stage 5. “Unable to stage” was set to stage 5 to capture all possible HAPI irrespective of the admission stage. On the basis of NPIAP documentation [20], deep tissue injury was either stage 3 or 4 PI. Therefore, to allow exclusion from the HAPI criteria because of stage improvement during the stay, we coded deep tissue injury as stage 4 at admission and stage 3 at discharge.

We excluded the stays that did not meet the common inclusion criteria. The common inclusion criteria across the four definitions were as follows: (1) presence of at least one clinical note, (2) documented discharge time as after admission time, (3) the patients aged <15 years, and (4) no admission documentation of a PI. *EHAPI* excluded patients who died in the hospital. We excluded deceased patients for three reasons: (1) adherence to CMS guidelines (including the need for a discharge PI stage that is not available in deceased patients), (2) potential bias of the computational model toward learning characteristics of deceased patients instead of HAPI, and (3) weakness and fragility in patients who have terminal illness result in PI occurrence and do not reflect poor nursing care quality. We conducted an experiment that included deceased patients and observed that some deceased patients were classified as HAPI when they were not HAPI cases.

**Figure 2:**
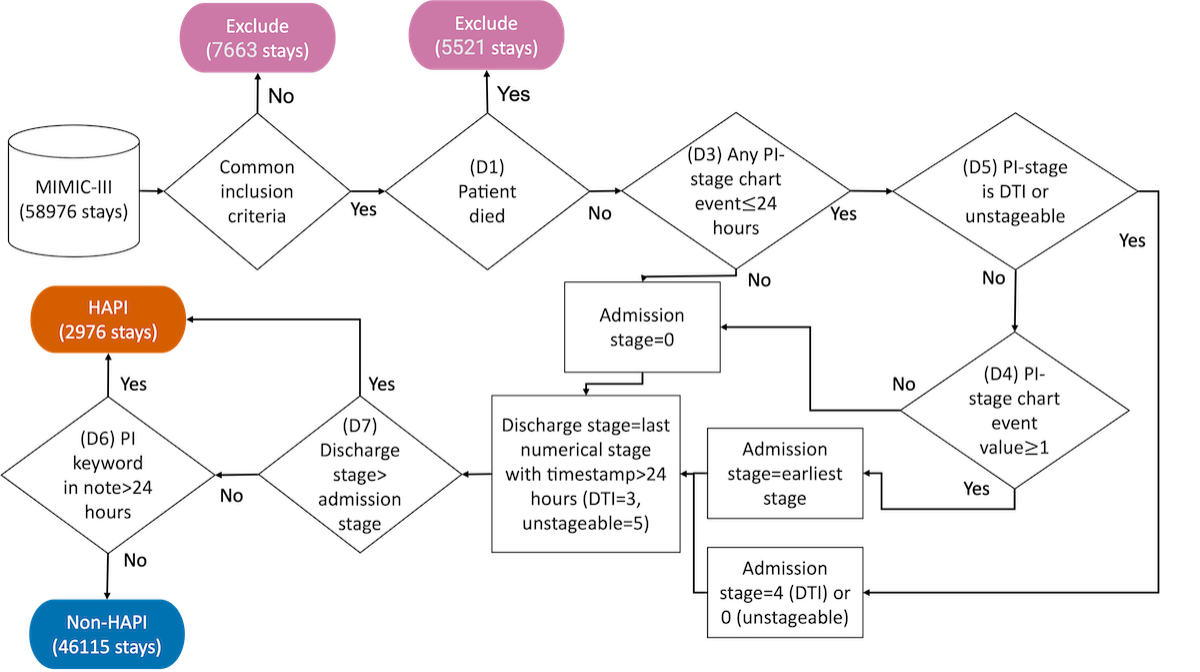
Flowchart for the Emory hospital-acquired pressure injury (*EHAPI*) definition. Common inclusion criteria across existing definitions and *EHAPI* are the presence of notes, patients aged 15 years and older, discharge time after admission time, and no pressure injury (PI) diagnosis on admission. D: dimension; DTI: deep tissue injury; HAPI: hospital-acquired pressure injury; MIMIC III: Medical Information Mart for Intensive Care III.

We found that some HAPI cases lacked PI staging events and yet contained PI keywords in their notes. Thus, the *EHAPI* also checked the PI-related keywords that occurred in notes later than 24 hours after admission. We scanned all stays for PI-related keywords mentioned in notes later than 24 hours after admission; if present, we considered these cases to be HAPI cases. We used negation detection and analyzed the notes of these cases to ensure that the keywords were not spurious (Multimedia Appendix 1). Other stays constituted the control group. Figure 2 provides the flowchart for the *EHAPI* case definition process.

To ensure the generalizability of the *EHAPI* definition, Multimedia Appendix 1 shows the HAPI-related SNOMED and ICD-10 codes used in many clinical information systems. However, the keywords for notes may need to be tailored to each hospital system. For details of the PI lists, keywords, and mappings in MIMIC-III used for the *EHAPI*, CANTRIP, Cramer, and Sotoodeh definitions, we refer the reader to Multimedia Appendix 1.

### Assessing Impact of HAPI Labels on Classification Performance

We compared the 3 existing HAPI definitions for HAPI classification in MIMIC-III with our *EHAPI* definition. One systematic review [21] study looked at data-driven models for PI prediction and risk assessment and concluded that many of these predictive models were difficult to compare because they were not externally validated and did not use the same data set.

#### Event Time Stamp Definition

For each hospital stay, we identified an event time stamp for feature construction. The idea is that for HAPI cases, HAPI-related information is not directly or indirectly present in the features (ie, target or label leakage). Similarly, for non-HAPI cases, we prevented biasing the classifier from predicting longer note durations that would be associated with non-HAPI stays. The event time stamp for HAPI cases is the time of the first PI stage assessment that occurred later than 24 hours after admission. The assessment is the earliest time stamp of either the PI staging chart event or the mention of any of the defined PI keywords in the notes.

For control stays, we matched the non-HAPI duration distribution with the HAPI duration distribution. We modeled the duration of the notes in case stays (the time difference between the earliest note and event time stamp) as a random variable. We used the *scikit-learn* [22] package to learn the density distribution for this random variable (ie, the estimated distribution with the smallest chi-square score). We then sampled the duration from this estimated distribution for the allowed note length for control stays. Each sampled duration pairs with a true duration length by preserving the ranked order (ie, the fifth smallest duration of sampled length and true length paired with each other). The minimum sampled length and true duration length then serve as the event time stamp (ie, earliest note+sampled length) for the control stay. Thus, if the sampled event time stamp exceeds the stay duration, then the event time stamp is the original stay discharge time.

#### Notes for HAPI Classification

Hospital stay features are based on patient notes. Machine learning models then only use notes with a time stamp before the event time stamp, or notes of interest, as features. If there were no notes of interest, the stay was excluded from the experiments. The notes of interest were then concatenated into a single document. This minimized the potential for label or target leakage, where HAPI-related information was directly or indirectly present in the features. For instance, defined PI-related keywords do not appear in the concatenated document. Similarly, the feature construction excludes notes after the first staging assessment, thereby preventing implicit PI-related words. Thus, feature construction excludes all elements discussed in the definition of the HAPI.

#### Classifiers

We chose 2 classifiers to demonstrate that the relative impacts of HAPI labels from the case or control definitions are independent of classifier choice. We chose gradient boosting, a tree-based classifier, and a sequential neural network–based classifier. The latter consisted of input word embedding learned from the features of each definition, a global max pooling layer, and several dense layers. The selected classifiers offer superior performance compared with other tested classifiers (ie, decision tree, logistic regression, support vector machine, multilayer perceptron, random forest, or AdaBoost).

The term frequency–inverse document frequency vector of the abridged notes (described in the aforementioned section) is the feature vector of each stay for the tree-based classifier (with a 5000-word vocabulary). The sequential neural network model uses a sequence of 800 words for each document. The 4 different HAPI definitions (ie, CANTRIP, Cramer, Sotoodeh, and *EHAPI*) used the same features to yield unbiased model performance comparisons with different definitions.

#### Train-Test Compositions and Evaluation Metrics

**Figure 3:**
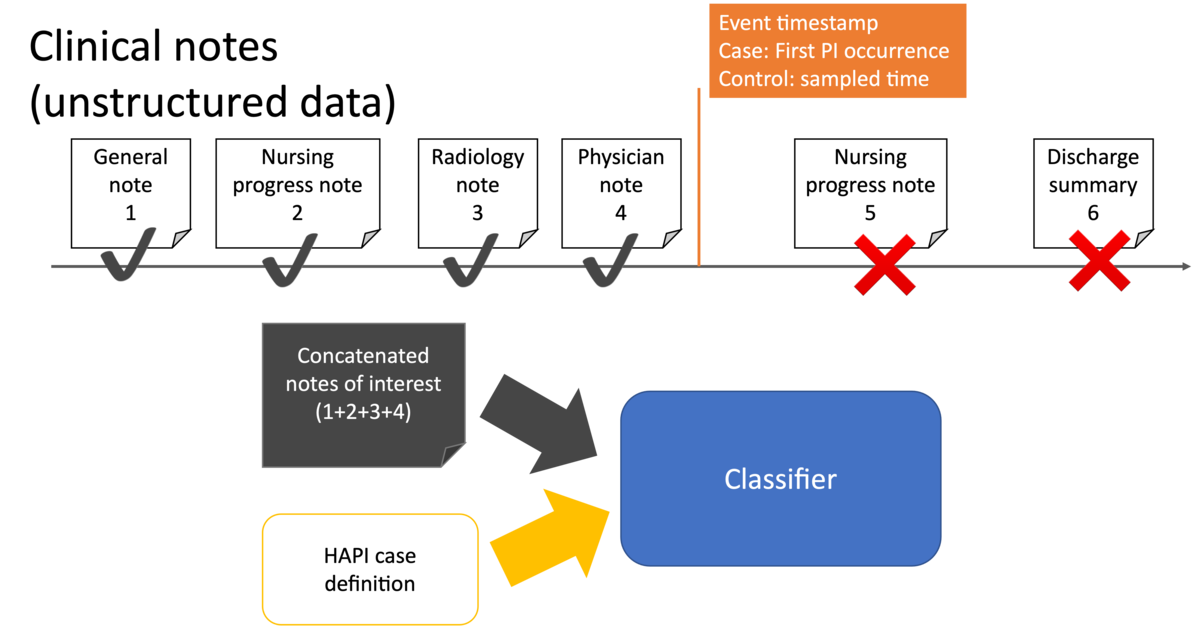
An illustrative example of the feature construction used to train the classifier including determination of the notes of interests using the event time stamp. Feature construction excludes notes 5 and 6 since they are after the event time stamp. HAPI: hospital-acquired pressure injury; PI: pressure injury.

Because HAPI criteria differed across definitions, the samples for the prior papers were different (eg, *EHAPI* discards deceased patients, but others have it either as a case or control). Nevertheless, there were considerable case overlaps across the samples (Figure 3). For a valid comparison, we created 10 different test sets consisting of three parts: (1) consensus HAPI case stays where the definitions agreed, (2) randomly subsampled consensus HAPI control stays, and (3) manually annotated stays where the definitions disagreed. For the latter stays, our nursing experts, a coauthor (WZ), and her nurse colleague (Deborah Silverstein, DNP) assessed and labeled 97 patient stays for HAPI based on the EHR data. The 97 stays constructed each constituent subset proportional to the total size of the differently labeled stay subset (Table S1 in Multimedia Appendix 1). Annotation relies not only on nursing guidelines but also on nursing experience and case discussion between the 2 nurses. Furthermore, one of our nurse annotators (Deborah Silverstein, DNP) was unaware of the *EHAPI* criteria and labeled the samples from a clinical practitioner’s perspective. Out of the 97 admissions, our nursing experts marked 19 as HAPI.

The manually annotated subset was augmented with consensus stays. There were 219 HAPI cases identified by the 4 definitions, which were included in the 10 test samples. The remaining 3620 non-HAPI stays were randomly sampled from the 41,241 admissions where all 4 definitions agreed on the label. Each test set contained 3936 stays and 7% HAPI prevalence. The main difference between each test set was the 3620 randomly sampled non-HAPI consensus stays, as the 97 manually annotated stays and 219 consensus HAPI cases were present in all test sets.

For each definition, the training samples were the remaining eligible stays that were not in the shared test set. As an example, because CANTRIP did not exclude stays with deceased patients, we included these in the training sample. The training labels were set using definition-specific HAPI criteria. Therefore, although the test samples and labels were the same, the classifier trained for each definition had a different training set and definition-specific label. Figure 3 illustrates the overall process for training the classifier, including the feature construction and label determination.

For the experiments, 5-fold cross-validation of the training set determined the best classifier hyperparameters, as shown in Table S2 in Multimedia Appendix 1. The test performance was the average of 10 different test and training data partitions. Given the unbalanced classes, we report both area under the precision-recall curve (AUPRC) and area under the receiver operating characteristic curve (AUROC).

### Ethical Considerations

The patients were not explicitly recruited to acquire the data used in this work. The MIMIC-III data set has been deidentified through elimination of attributes revealing patients’ identity.

Approval for data collection, processing, and release for the MIMIC-III database was granted by the Institutional Review Boards of the Beth Israel Deaconess Medical Center (Boston, United States) and Massachusetts Institute of Technology (Cambridge, United States).

## Results

### Consistency of MIMIC-III Data Sources for PI Hospital Stays

Even without consideration of patient attributes or timing, evidence shows conflicts among data sources for identifying PI case hospital stays in MIMIC-III. Figure 1 presents an UpSet plot that summarizes the intersection of PI-related information across the 4 data sources (ie, procedure codes, diagnosis codes, notes, and chart events) for stays with at least one data source indication of PIs (7908 total stays). The data source bar charts (bottom left side) plot the cardinality (or size) of the number of stays with the data source indication. Chart events were the most common indicator (6243/7908, 78.95%), whereas procedure codes only appeared in 3.53% (279/7908) of the stays. The bar charts along the x-axis plot the size of the intersections among the observed set combinations. The results demonstrate limited consensus among the data sources, as only 0.25% (20/7908) of the stays had PI documentation across all data sources. Even agreement among ≥3 data sources was relatively low, with 945 stays (927+9+9). This can be contrasted with 49.34% (3902/7908) and 9.71% (768/7908) of the stays containing only an indication in the chart events and notes, respectively.

### Analyzing Differences in HAPI Case Definitions

On the basis of the 4 HAPI case definitions, there are 8 dimensions in which the criteria diverge. The exclusion criteria encompass deceased patients (D1), minimum age (D2), and the amount of time to ascertain PIs on admission (D3). The determination of HAPI includes the minimum PI stage (D4), consideration of deep tissue injury or unstageable events (D5), use of PI-specific keywords in the notes (D6), calculation of deteriorating or new PIs (D7), and use of ICD-9 codes (D8). Table 1 summarizes the decisions along these 8 dimensions for the 4 different definitions. As can be observed from the table, *EHAPI* definition excludes the deceased entirely from case or control and ascertains whether the PI deteriorated or newly developed. Both the Cramer and Sotoodeh definitions yielded substantially lower estimates of HAPI prevalence, whereas CANTRIP had the highest prevalence at 8.46% (4261/50,376).

**Figure 4:**
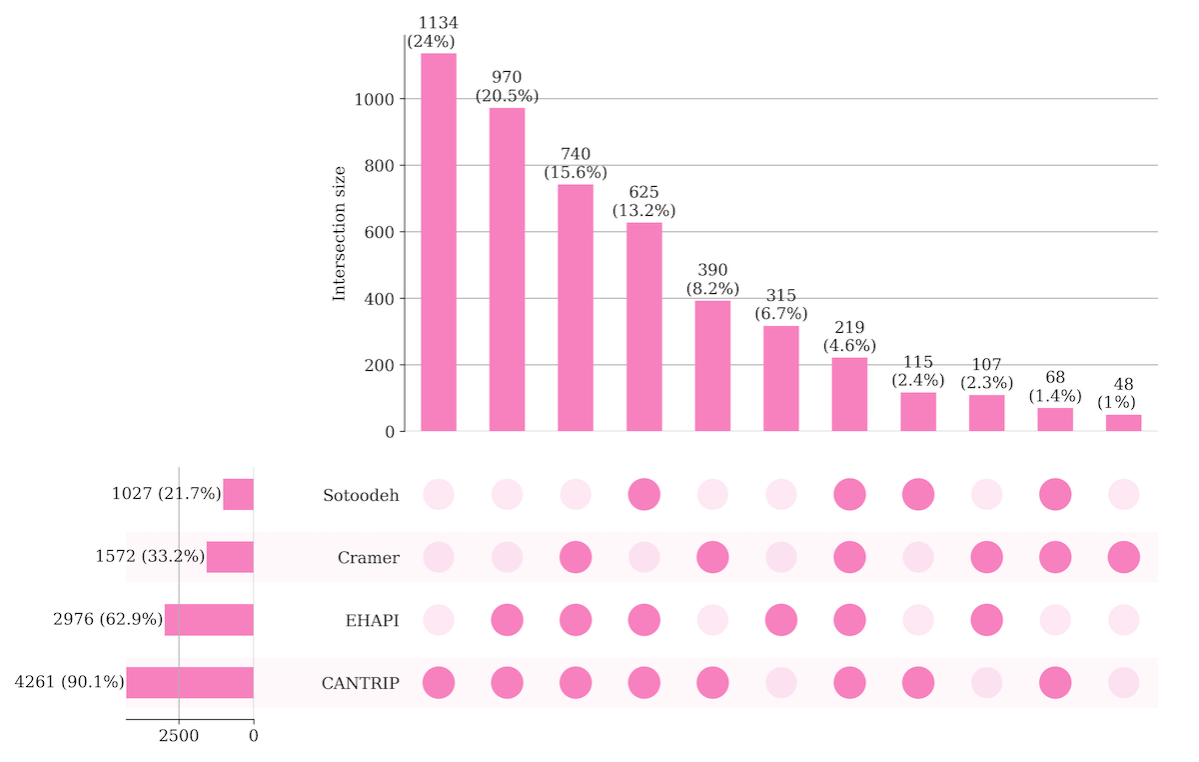
Overlap of hospital-acquired pressure injury (HAPI) stays across the 4 definitions. CANTRIP: recurrent additive network for temporal risk prediction; *EHAPI*: Emory hospital-acquired pressure injury.

An UpSet plot capturing the overlap between HAPI stays across the 4 definitions is shown in Figure 4. Only 4.63% (219/4731) of HAPI stays shared 4 definitions. CANTRIP had the highest number of unique positive stays (n=1134), arising from considering PI stages above 1 and deep tissue injury and unstageable events as positives. We observed 315 stays unique to *EHAPI*, attributed to the cutoff period (24 vs 48 with CANTRIP). *EHAPI* had the highest overlap, 53.98% (2554/4731) with CANTRIP, followed by Cramer at 22.53% (1066/4731) and Sotoodeh at 17.84% (844/4731). The details of the number of PIs identified using notes, staging, and ICD-9 codes for each definition are provided in Multimedia Appendix 1.

**Table 1:** Definition properties and compositions along the 8 criteria dimensions (Ds).

Definition	D1^a^	D2^b^	D3^c^	D4^d^	D5^e^	D6^f^	D7^g^	D8^h^	Cases, n (%)
*EHAPI*^i^ (n=44,823)	Yes	15	24 h	2	Yes	Yes	Yes	No	2976 (6.64)
CANTRIP^j^ (n=50,376) [10]	No	15	48 h	1	Yes	Yes	No	No	4261 (8.46)
Cramer (n=50,276) [6]	No	18	24 h	2	No	No	No	No	1572 (3.13)
Sotoodeh (n=50,276) [9]	No	18	N/A^k^	N/A	N/A	Yes	No	Yes	1027 (2.04)

^a^D1 denotes the decision of whether to exclude deceased.

^b^D2 refers to the minimum age in years.

^c^D3 indicates the cutoff period for determining preexisting pressure injury (PI).

^d^D4 characterizes the minimum numerical PI stage.

^e^D5 signifies whether deep tissue injury or unstageable PI staging chart events are hospital-acquired pressure injury (HAPI).

^f^D6 represents whether PI keywords present in notes are considered an HAPI event.

^g^D7 designates whether the criteria captured worsening or newly developed PI.

^h^D8 captures whether International Classification of Diseases 9 codes use HAPI for identification.

^i^*EHAPI*: Emory hospital-acquired pressure injury.

^j^CANTRIP: recurrent additive network for temporal risk prediction.

^k^N/A: not applicable.

### Impact of HAPI Labels on Classification Performance

We evaluated the performance of the gradient boosting and sequential neural network classifiers trained on labels determined by the 4 HAPI definitions by using AUPRC and AUROC for each case. Table 2 presents the results for the 10 described test sets. Table S3 in Multimedia Appendix 1 summarizes the performance based on the test label source (ie, nurse or consensus). Classifiers trained on the *EHAPI* criteria performed better than those trained on other 3 criteria with an improvement in AUROC up to 0.03 and in AUPRC up to 0.11.

A 1-sided paired *t* test (1-tailed) between *EHAPI* and the next best performing definition (CANTRIP) resulted in a *P* value of <.001 for AUPRC and AUROC for the better-performing gradient boosting classifier and machine epsilon for other classifiers and definitions (except neural networks and CANTRIP), demonstrating the merits of the *EHAPI* definition. Further analysis of the models’ performance stability and the most important words in each setting are provided in Figure S6 and Table S4 in Multimedia Appendix 1. A GitHub repository [23] contains scripts for these experiments, the generation of the stay labels, and the other presented results.

**Table 2:** Classifiers’ performance for the 4 hospital-acquired pressure injury definitions in Medical Information Mart for Intensive Care III over 10 test sets. The results represent the average across test sets with the SD in parenthesis and the *P* value.

Definition	Gradient boosting				Neural networks			
	AUPRC^a^		AUROC^b^		AUPRC		AUROC	
	Mean (SD)	*P* value	Mean (SD)	*P* value	Mean (SD)	*P* value	Mean (SD)	*P* value
*EHAPI* ^c^	0.46 (0.015)	N/A^d^	0.90 (0.003)	N/A	0.44 (0.015)	N/A	0.88 (0.004)	N/A
CANTRIP^e^ [10]	0.44 (0.017)	≤.001	0.89 (0.003)	≤.001	0.41 (0.010)	≤.001	0.88 (0.005)	.02
Cramer [6]	0.35 (0.015)	≤.001	0.87 (0.005)	≤.001	0.38 (0.022)	≤.001	0.86 (0.006)	≤.001
Sotoodeh [9]	0.33 (0.015)	≤.001	0.87 (0.004)	≤.001	0.35 (0.015)	≤.001	0.86 (0.006)	≤.001

^a^AUPRC: area under the precision-recall curve.

^b^AUROC: area under the receiver operating characteristic curve.

^c^*EHAPI*: Emory hospital-acquired pressure injury.

^d^N/A: not applicable.

^e^CANTRIP: recurrent additive network for temporal risk prediction.

## Discussion

### Principal Findings

Given the low concurrence of PI between data sources, any HAPI classification requires careful reconciliation of conflicts between data sources. On the basis of discussions with our nursing collaborators (WZ, RN, PhD; Deborah Silverstein, RN, DNP; and RLS, RN, DNP), we prioritized data source reliability as (1) chart events, (2) notes, and (3) diagnosis codes. Charting events were least likely to have a false positive and had better coverage than the other 2 data sources. The nurses indicated that PI indications from notes have false positives, as keywords are preceded by a negative word (ie, no PI), or denote suggestions for PI prevention. Diagnosis codes include only the most prominent diagnoses and might include diagnoses of earlier admissions. In addition, their lack of time stamps prevents investigation of the deterioration condition of the HAPI. Because procedure codes are not specific and inconsistent with other PI sources, we excluded them from the *EHAPI* definition.

As shown in Table 2, the classifiers trained using the *EHAPI* definition achieved the best performance. Moreover, the AUROC of the resulting classifiers from 4 definitions were consistently high (≥0.86). The high AUROC is consistent with the CANTRIP results (AUROC of 0.87) [10] and Sotoodeh results (AUROC of 0.95) [9]. However, the AUPRC remains unacceptable, with the highest performance achieved by gradient boosting (0.46). These values are consistent with the existing literature, as CANTRIP reported precision and recall of 0.42 and 0.71, respectively [10], and Cramer reported precision and recall of 0.09 and 0.71, respectively [6]. This illustrates that to identify the HAPI cases, the computational model generates a sizeable portion of false positives.

### Limitations and Future Work

CMS-defined guidelines specify that HAPI are only newly developed, unhealed, or deteriorated PIs. Unfortunately, this involves matching admission and discharge PIs, as a patient may be admitted with >1 PI and discharged with more or fewer PIs. The deterioration condition describes each PI individually. However, given the limited data in the event table of MIMIC-III, our case criteria assume that stays are associated with only one PI. Further analysis of multiple possible PI locations yielded better grouping. However, unless skin assessments at admission and discharge are documented in a structured format, matching PIs is difficult. Ideally, the “deteriorated PI” criterion applies to positive PI samples using patient notes as well. However, information on the PI stage is difficult to obtain from notes and, thus, is not implemented in the current case definition. We plan to study the HAPI in other data sets that have better PI documentation practices to fully understand the impact of multiple PIs.

Another limitation of our study is the use of a simple negation detection algorithm to identify false positives occurring with positive PI mentions in the clinical notes. The keyword list disregarded structure matches such as “bedsore: none,” and the negation detection mainly captures instances of text that mentioned “no bedsore observed.” However, instances of negation in more complex textual descriptions may be missed, thus creating false positives in the identified 2976 HAPI stays. A manual inspection of the 1175 case stays labeled through the PI keyword mentions route is left for future work.

Enhancing the manually labeled samples in the 10 test sets beyond the 97 randomly selected ones is another avenue for future research. The small curated set was not large enough for stand-alone analysis, as it yielded large performance variations across the test sets. Unfortunately, it was labor intensive for our nursing annotators to annotate the samples; thus, further annotation is beyond the scope of this work.

In addition, we note that our assessment of the impact of the HAPI definition is based only on MIMIC-III. Furthermore, MIMIC-III contains data collected only in critical care settings. To better understand the performance implications of the HAPI definition, applying implications to other settings, such as the general care units, as well as other health care systems, is needed. We plan to apply these *EHAPI* criteria to define HAPI in more data sets.

In addition to the focus on critical care stays, the MIMIC-III has unique demographic characteristics, such as predominantly Caucasian. We plan to test the generalizability and impact of the *EHAPI* case definition against more data sets with diverse demographics including higher percentages of African American, Asian, and Hispanic individuals or different insurance compositions.

A recent systematic review on the utility of decision support systems for PI management concluded that their adoption in practice has clinical significance in terms of reducing PI incidence and prevalence, but statistical significance was not observed [24]. This emphasizes the importance of studying practical challenges in the adoption of data-driven PI methods by nurses. Moreover, the practical deployment of a computational model necessitates a higher AUPRC to prevent false alarms. Thus, an open question is whether the integration of other patient information in addition to clinical notes, such as physiological measurements, patient demographics, and medications, yields better predictive performance.

### Conclusions

An accurate definition of HAPI based on clinical data is critical for automating nursing quality metrics and for valid comparisons of HAPI machine learning models. However, one of the major challenges is the inconsistency of the PI indicators across various data sources. We demonstrate the lack of congruency between the 3 existing HAPI definitions for MIMIC-III and highlight the gaps between each definition and the CMS and AHRQ regulatory guidelines. We then created a refined definition, the *EHAPI*, that more closely reflects the regulatory guidelines. Our experimental results using 2 different classifiers illustrate the impact of the definition on the predictive performance when evaluated on an unseen combination of a small, manually labeled set by 2 nurse annotators and a random sample of the consensus set (ie, all 4 definitions agree on the labels). This reinforces the need for a high-quality standardized HAPI definition, as the *EHAPI* achieves a better predictive performance across multiple test sets.

## Appendix

Multimedia Appendix 1 The supplementary material containing the Medical Information Mart for Intensive Care III details for the Emory hospital-acquired pressure injury definition, additional comparisons between the 4 case definitions, and further experimental results related to this study.

## Abbreviations

AHRQ: Agency for Healthcare Research and Quality
AUPRC: area under the precision-recall curve
AUROC: area under the receiver operating characteristic curve
CANTRIP: recurrent additive network for temporal risk prediction
CMS: Center for Medicare and Medicaid
DOE: date of event
EHAPI: Emory hospital-acquired pressure injury
EHR: electronic health record
HAPI: hospital-acquired pressure injury
ICD: International Classification of Diseases
MIMIC-III: Medical Information Mart for Intensive Care III
NPIAP: The National Pressure Injury Advisory Panel
PI: pressure injury

## Data Availability

Medical Information Mart for Intensive Care III (MIMIC-III) data can be downloaded from the PhysioNet webpage after completing the required Collaborative Institutional Training Initiative, data or human subjects research training. The scripts used to preprocess the data and obtain the presented results can be found in the GitHub repository.
