# Supplementary material for: A Comprehensive and Improved Definition for Hospital-Acquired Pressure Injury Classification Based on Electronic Health Records: Comparative Study
Source: JMIR Med Inform. 2023 Feb 23;11:e40672. doi: 10.2196/40672 (PMC9999254; doi:10.2196/40672)
Supplement: Multimedia Appendix 1 [file medinform_v11i1e40672_app1.docx]

# APPENDIX

This is a Multimedia Appendix to a full manuscript published in the J Med Internet Res. For full copyright and citation information see <http://dx.doi.org/10.2196/jmir.40672>

**Section S1 Details of MIMIC-III data sources for PI case hospital stays**

Here we explain the utility of different data sources tables in MIMIC-III for identifying PI hospital stays. The lists mentioned can be found in Section S4.

Chart events**:** In MIMIC-III, medical events are stored in *‘chartevents’* table*.* Each timed event in this table has an ‘itemid’ paired with a medical concept. We filtered all PI-stage-related events with ‘itemid’s in List 1 as they are paired with PI staging concepts. For example, ‘itemid’ 224970 is paired with ‘pressure ulcer stage 7’. This list was obtained from the union of the CANTRIP and Cramer definitions which used ‘chartevents’ to determine the label. As an example, for a specific clinical event with ‘itemid’ 224970, the value attribute is ‘unable to assess.’ Next, we assigned a stage number to events based on their ‘value’ attribute and the mapping based on [19], provided in the supplementary materials. There is also a ‘valuenum’ integer attribute for stage, but many values were missing. A hospital stay may have multiple PI staging events.

Discharge ICD9 codes: Discharge codes in MIMIC III are stored in ‘ICD’ table. We labeled each hospital stay that used ICD9 codes in list 2 (based on [20]) to indicate a PI case. Unlike a previous study [17], we excluded ICD9 codes mapping to non-pressure injuries in the ICD10 system. Only Sotoodeh [6] used ICD9 codes for labeling positive samples.

Notes: Using the ‘Noteevents’ table in MIMIC-III, notes of each stay were checked for keywords or regex patterns that indicated ulcers or sores (list 3). This list covers most terms used to refer to PI, including common misspellings, and disregards structural matches, i.e., “bedsore: none”. The keywords were obtained from both the CANTRIP and Sotoodeh definitions.

**Section S2 The procedure for extraction of cases and control groups based on existing HAPI definitions in MIMIC-III**

The flow diagrams for the three different definitions used as comparisons in the experiments. Common inclusion criteria across all definitions includes: (1) patients contain at least one note; (2) patients are age 15 and above; (3) discharge time is after the admission time; and (4) no PI diagnosis at admission.

**
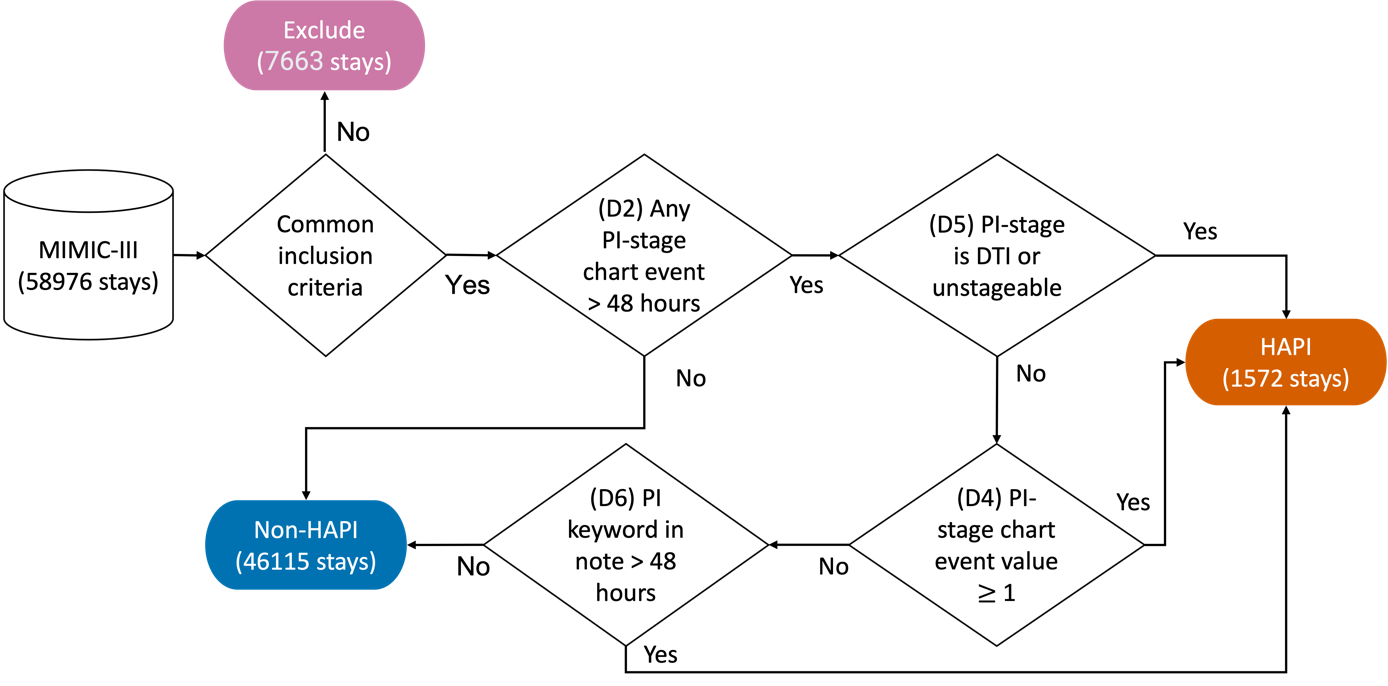
**

**Figure S1**. A flow diagram for the CANTRIP definition process.


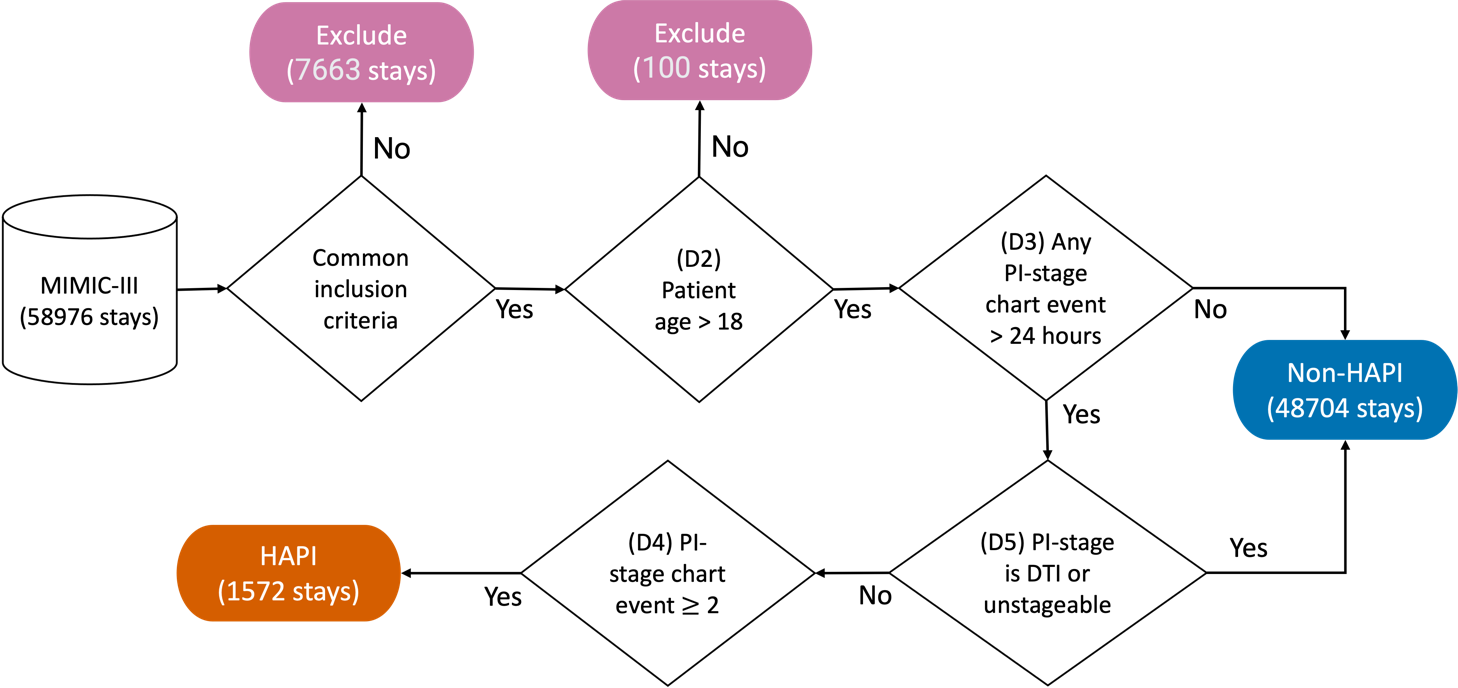


### **Figure S2**. A flow diagram for the Cramer definition process.

### **
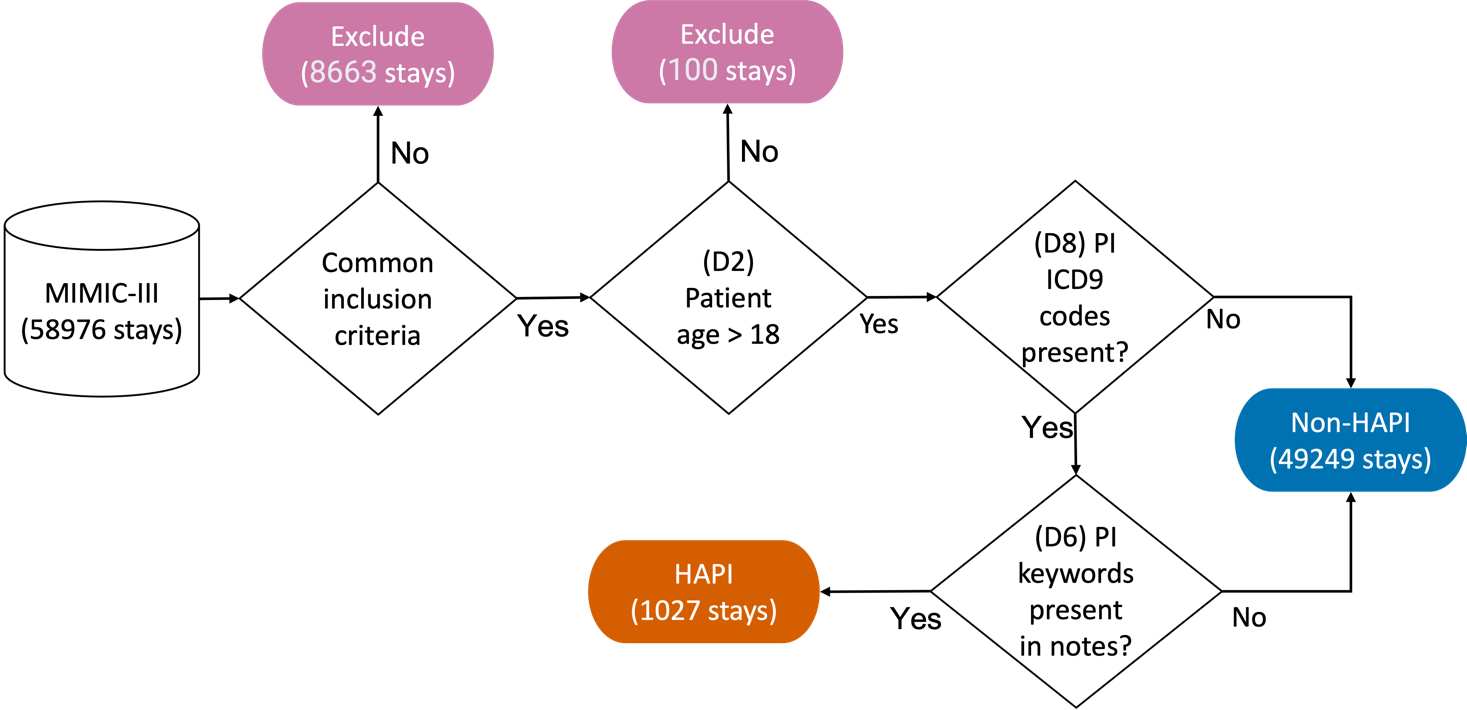
 Figure S3**. A flow diagram for the Sotoodeh definition.

**Section S3 Negation detection for excluding stays with negative mentions of PI**

For 1211 stays with no PI events in chartevents table, that had at least one of the keywords in List 3 in their notes at least than 24 hours after admission, and therefore could be included in *EHAPI*, we applied a negation detection step to exclude those stays with negative mentions of PI from this set. To mark negative PI mentions in the notes, we used two clinical text processing methods, Scispacy and NegEX. Initially Scispacy extracts all mentions of named entities including PI. NegEX package considers certain regular expressions that cover several negation patterns, filters out sentences that falsely appear to be negative, and sets the scope of the negation.

We then checked the explicitly negated notes resulting from the above procedure for negative mentions of any of the keywords in List 3 (i.e., no_bedsore). 36 stays included such negative mentions of PI, which we completely excluded from our study.

**Section S4 Lists used to extract relevant information from MIMIC-III tables**

#### PI-staging ‘itemid’s in ‘chartevents’ across Cramer, CANTRIP, and EHAPI:

####

| **Cramer** | **CANTRIP + EHAPI** |
| --- | --- |
| 551, 552, 553, 224631, 224965, 224966 | 551, 552, 553, 224631, 224965, 224966, 224967, 224968, 224969, 224970, 224971, 227618, 227619 |

#### List 2 PI-indicative ICD9 codes

[707, 707.1, 707.2, 707.3, 707.4, 707.5, 707.6, 707.7, 707.9, 707.11, 707.21, 707.22, 707.23, 707.24, 707.25]

#### List 3 keywords and regex patterns indicating PI

['(bed sore($|[^:]))', '(bed ulcer($|[^:]))', '(pressure sore($|[^:]))', '(pressure ulcer($|[^:]))', '(decub(\w*\s*) sore($|[^:]))', '(decub(\w*\s*) ulcer($|[^:]))']

#### List4 CPT codes for PI care

[11042, 11043, 11044, 15999, 97598, 97597, 16020, 16030, 15835, 15878, 15879, 27027, 27057]

## Section S5 PI stage-keyword mapping

*Stage 1:* 'Intact,Color Chg' - 'Red; unbroken' - 'Red, Unbroken'

*Stage 2:* 'Through Dermis' - 'Part. Thickness'- 'Partial thickness skin loss through epidermis and/or dermis; ulcer may present as an abrasion, blister, or shallow crater.'

*Stage 3:* 'Through Fascia' - 'Full Thickness' - 'Full thickness skin loss that may extend down to underlying fascia; ulcer may have tunneling or undermining.'

*Stage 4:* 'To Bone'- 'Full thickness skin loss with damage to muscle, bone, or supporting structures; tunneling or undermining may be present.'

Ambiguous stage*:* 'Deep tissue injury' - "Deep Tiss Injury" - 'Unable to assess; dressing not removed' - 'Unable to stage; wound is covered with eschar' - 'Unable to Stage.'

*Not indicating a definitive PI case:* Any other value not in the above categories.

**Section S6 HAPI related SNOMED and ICD10 codes**

| **SNOMED Name** | **SNOMED Code** | **ICD9 Code** | **ICD10 Code** |
| --- | --- | --- | --- |
| Pressure ulcer stage 1 | 421076008 | 707.21 | [L89.011, L89.311, L89.601, L89.141, L89.521, L89.211, L89.121, L89.91] |
| Pressure ulcer, unstagable | 399912005 | 707.25 | [L89.95] |
| Pressure ulcer stage 3 | 421927004 | 707.23 | [L89.513, L89.313, L89.143, L89.223, L89.103, L89.113, L89.603, L89.023] |
| Pressure ulcer of heel | 225561003 | 707.07 | [L89.602, L89.624, L89.601, L89.629, L89.603] |
| Pressure ulcer stage 4 | 420597008 | 707.24 | [L89.204, L89.624, L89.124, L89.324, L89.894, L89.144, L89.014, L89.514] |
| Pressure ulcer stage 2 | 420324007 | 707.22 | [L89.602, L89.312, L89.812, L89.522, L89.122, L89.42, L89.202, L89.002] |
| Pressure ulcer of buttock | 225563000 | 707.05 | [L89.311, L89.324, L89.313, L89.319, L89.312] |
| Pressure ulcer of elbow | 225559007 | 707.01 | [L89.011, L89.029, L89.014, L89.023, L89.002] |
| Pressure ulcer of ankle | 699211004 | 707.06 | [L89.513, L89.509, L89.521, L89.522, L89.514] |
| Pressure ulcer of hip | 699214007 | 707.04 | [L89.204, L89.223, L89.209, L89.211, L89.202] |
| Pressure ulcer of lower back | 699215008 | 707.03 | [L89.141, L89.143, L89.45, L89.144, L89.42] |
| Pressure ulcer of upper back | 699218005 | 707.02 | [L89.124, L89.129, L89.113, L89.122, L89.121] |

**Section S7 Distribution of manually labeled HAPI cases in different definitions**

| Chosen subset | # HAPI samples chosen | # samples in manually labeled HAPI samples | % of samples in manually labeled HAPI samples |
| --- | --- | --- | --- |
| EHAPI | 20 | 9 | 45% |
| CANTRIP-EHAPI | 25 | 5 | 20% |
| Cramer | 10 | 2 | 20% |
| CANTRIP | 30 | 2 | 7% |
| CANTRIP-Cramer | 12 | 1 | 7% |
| Total | 97 | 19 | NA |

We compared the ratio of the overlap of HAPI cases between each exclusive subset of definitions and the manually labeled HAPI cases, which shows the higher level of agreement of *EHAPI* with labels from nursing practitioners, compared to other definitions. Our nurse annotators labeled samples that not all four definitions agree on. The distribution of these chosen 97 samples across the subsets, is proportional to the total number of samples in each conflicting set. In Table S1 and Figure S4 we are showing the distribution of the 19 HAPI manually labeled cases across these sets. If the percentage of overlap is higher, it reflects that the criteria suggested by the definition(s) in that subset are closer to HAPI ground truth. In Figure S4 we can observe that for *EHAPI*, 45% of samples that were considered HAPI only by *EHAPI* were manually labeled positive and 20% of samples marked positive by both *EHAPI* and CANTRIP were also manually labelled positive.

### **Table S1.** Distribution of HAPI samples over conflicting subsets


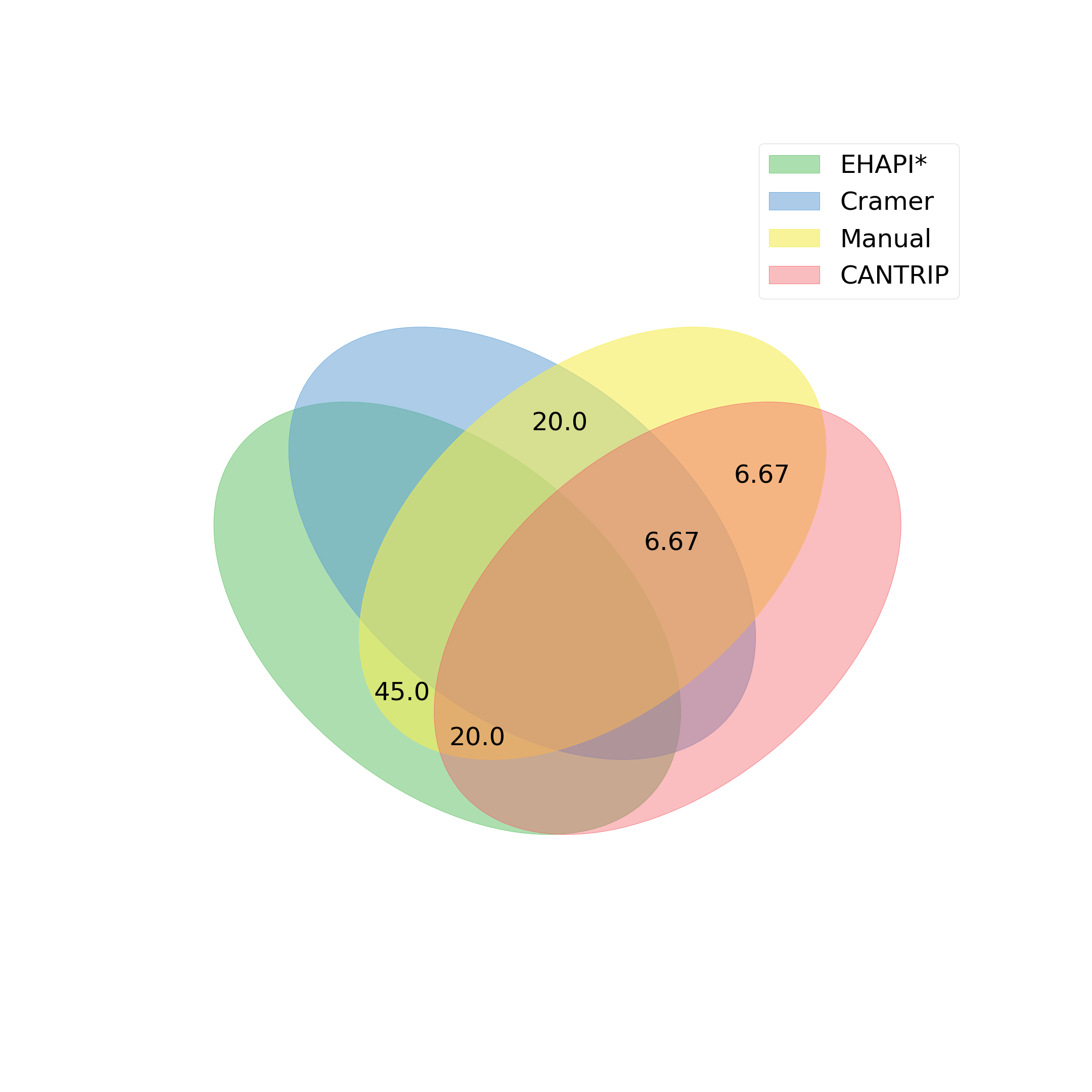


**Figure S4** – Distribution of 19 manually labeled HAPI positive cases over different definitions. The numbers reflect the percentages of agreement. For example, for 20% of the 19 cases only Cramer marked it as a positive, whereas for 45% of the 19 cases only EHAPI marked it as positive.

## Section S8 Hyperparameter tuning

We chose the best parameters for the classifier on training data for each definition within a partition by doing a five-fold cross-validation grid search from the values in Table S2.

| Parameter | Values |
| --- | --- |
| Number of estimators for gradient boosting classifier | [100,200,400, 800] |
| Batch size for sequential neural network classifier | [16,32,64,128] |

**Table S2**. Hyperparameter values for the classifiers used

**Section S9 The intersection of HAPI-positive populations from notes and events for different definitions**

Venn diagrams show the overlap of HAPI-positive cases based on staging data versus notes for the four definitions (Figure S5).

**
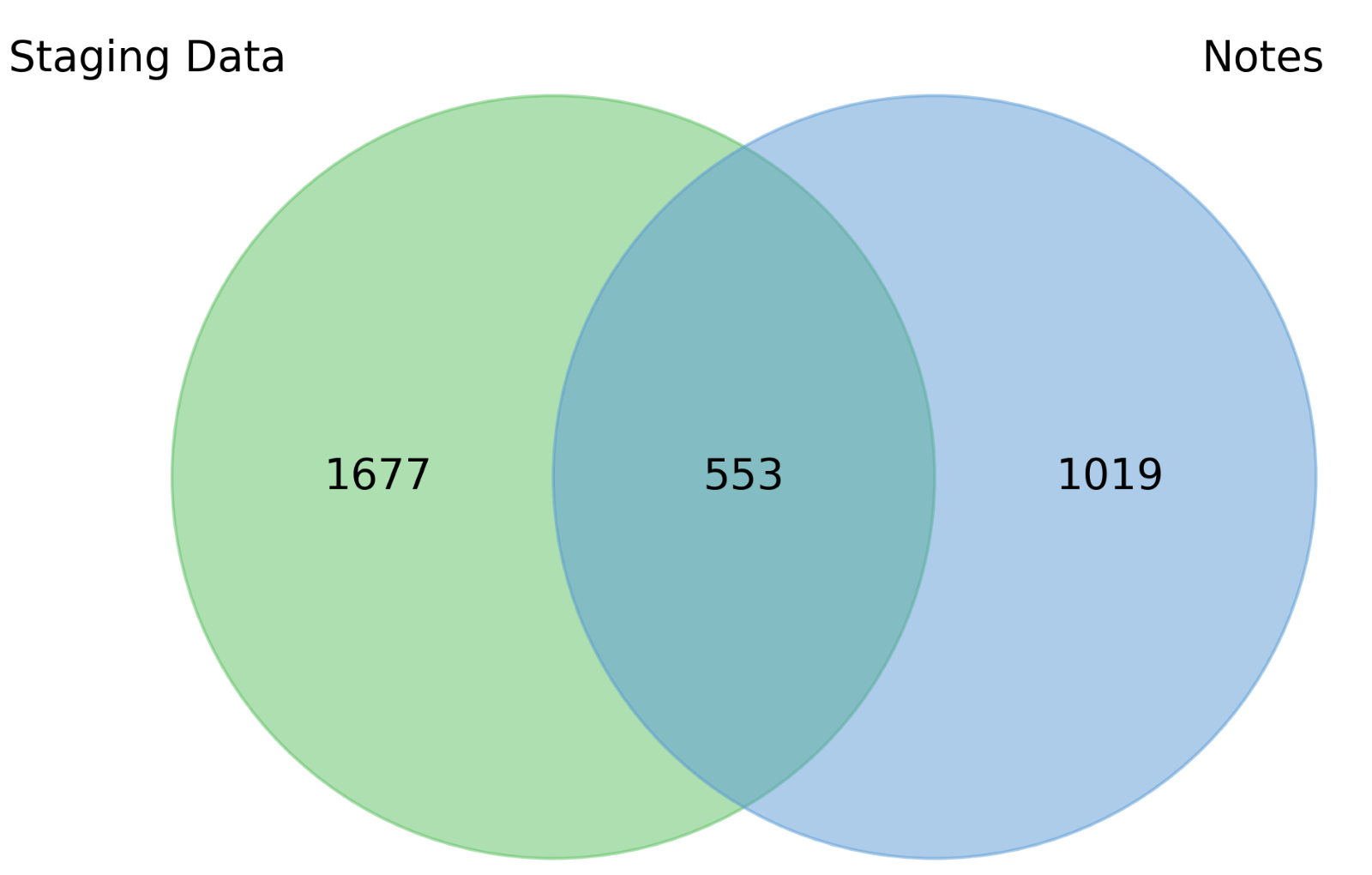
**

1. Cramer

**
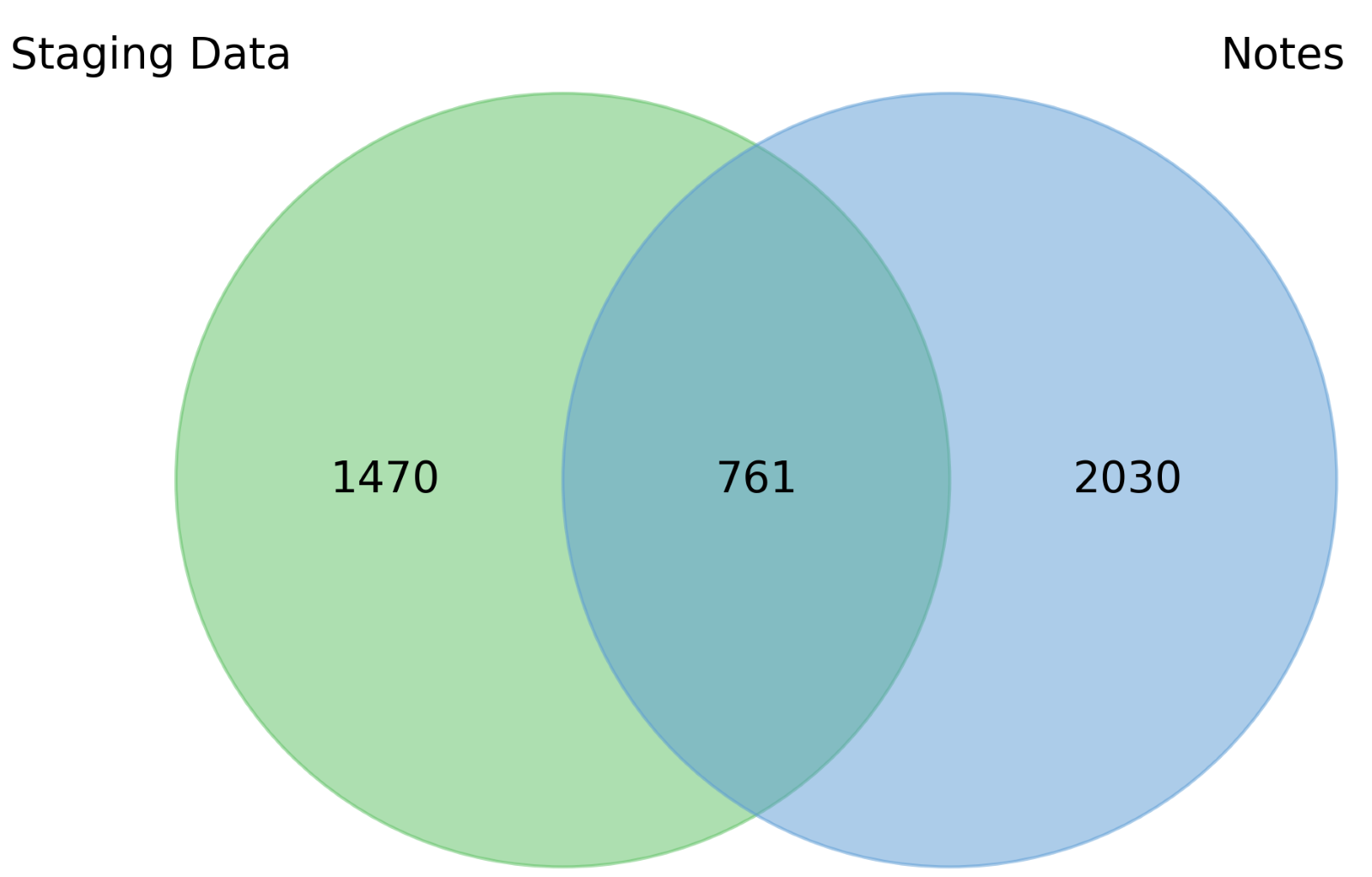
**

1. CANTRIP

**
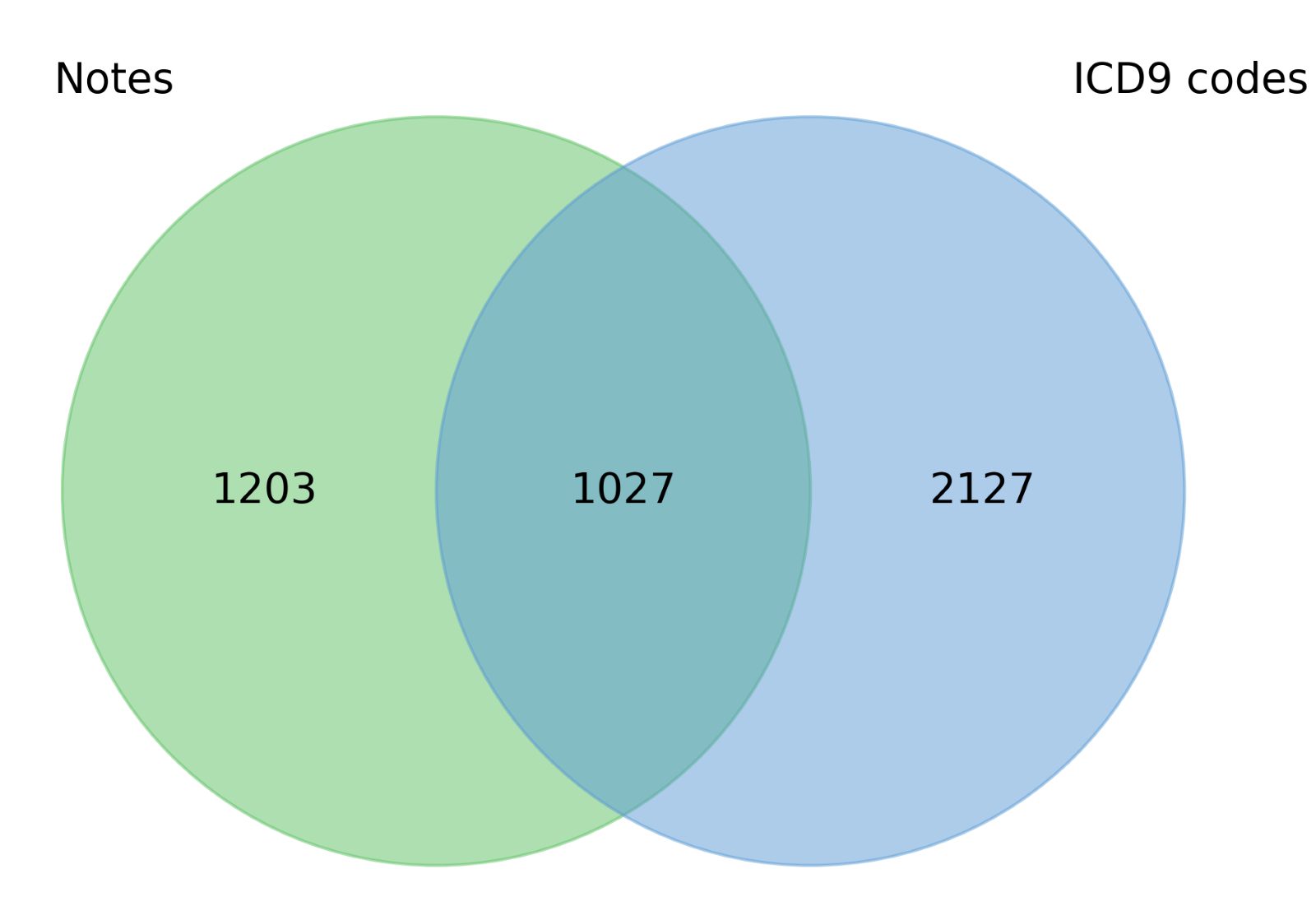
**

c)  Sotoodeh

**
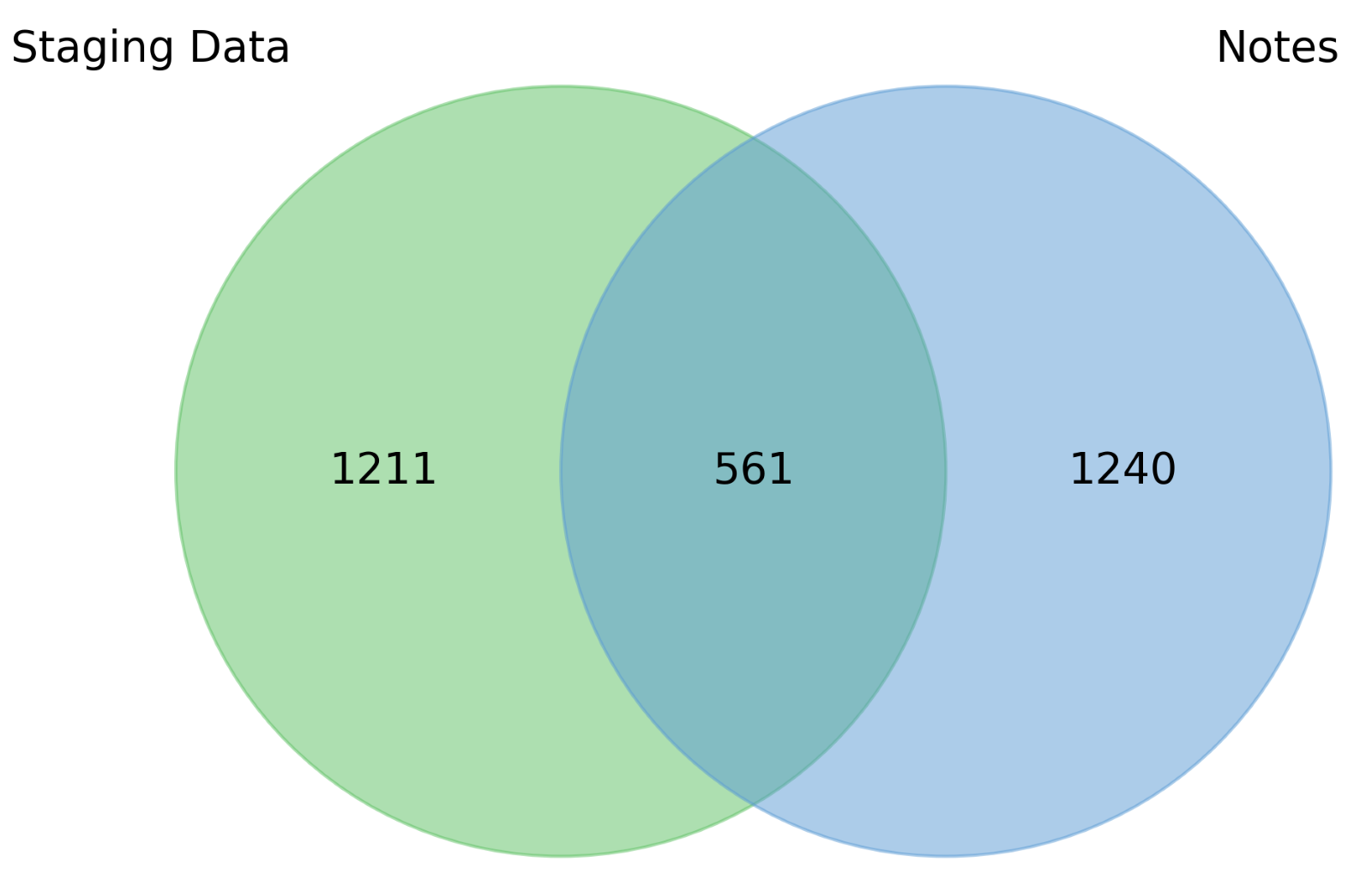
**

            d) EHAPI

**Figure S5.** Overlap of HAPI indicative stays in multiple sources in the four definitions.

## Section S10. Generalizability of models trained on the different definitions

In Figure S6, we show the violin plots of all definitions’ performance on 10 test sets across the 2 metrics and the 2 classifiers, to highlight that our definition not only on average perform better than the other definitions, but also has either equal or a smaller variance in performance in all 4 settings compared to the 3 different definitions.

**
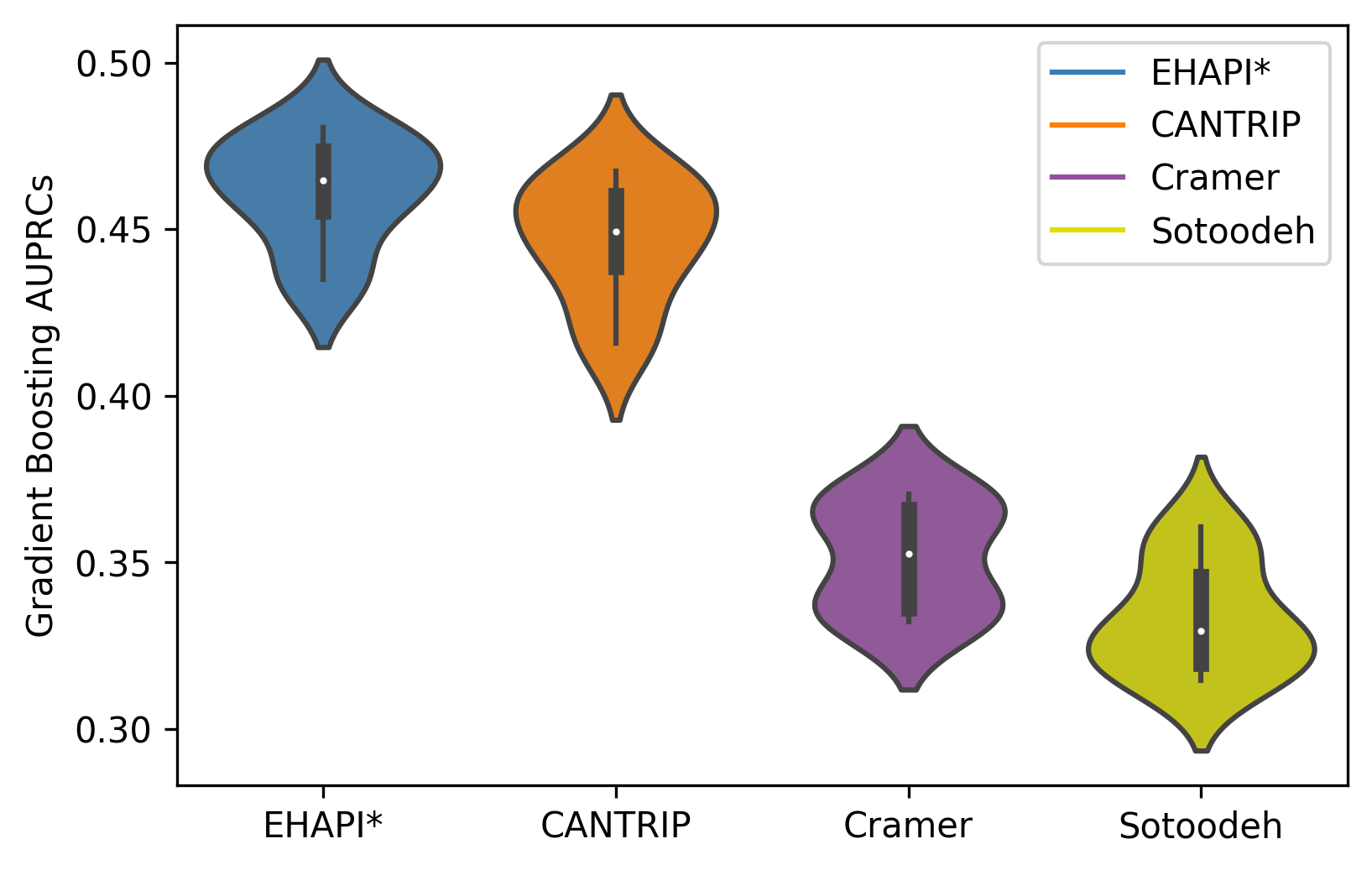
**

1. Gradient boosting- AUPRC

**
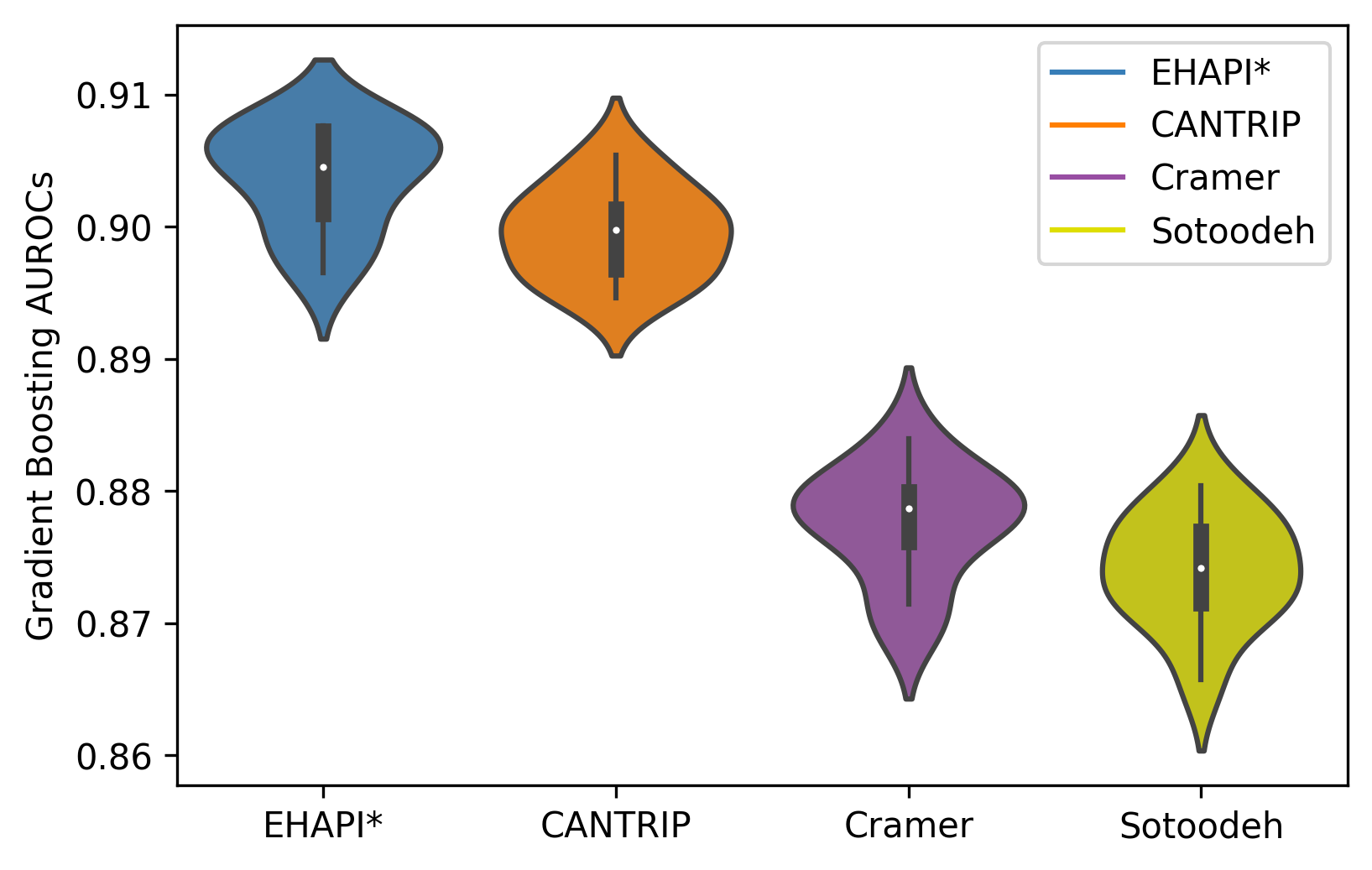
**

1. Gradient boosting- AUROC

**
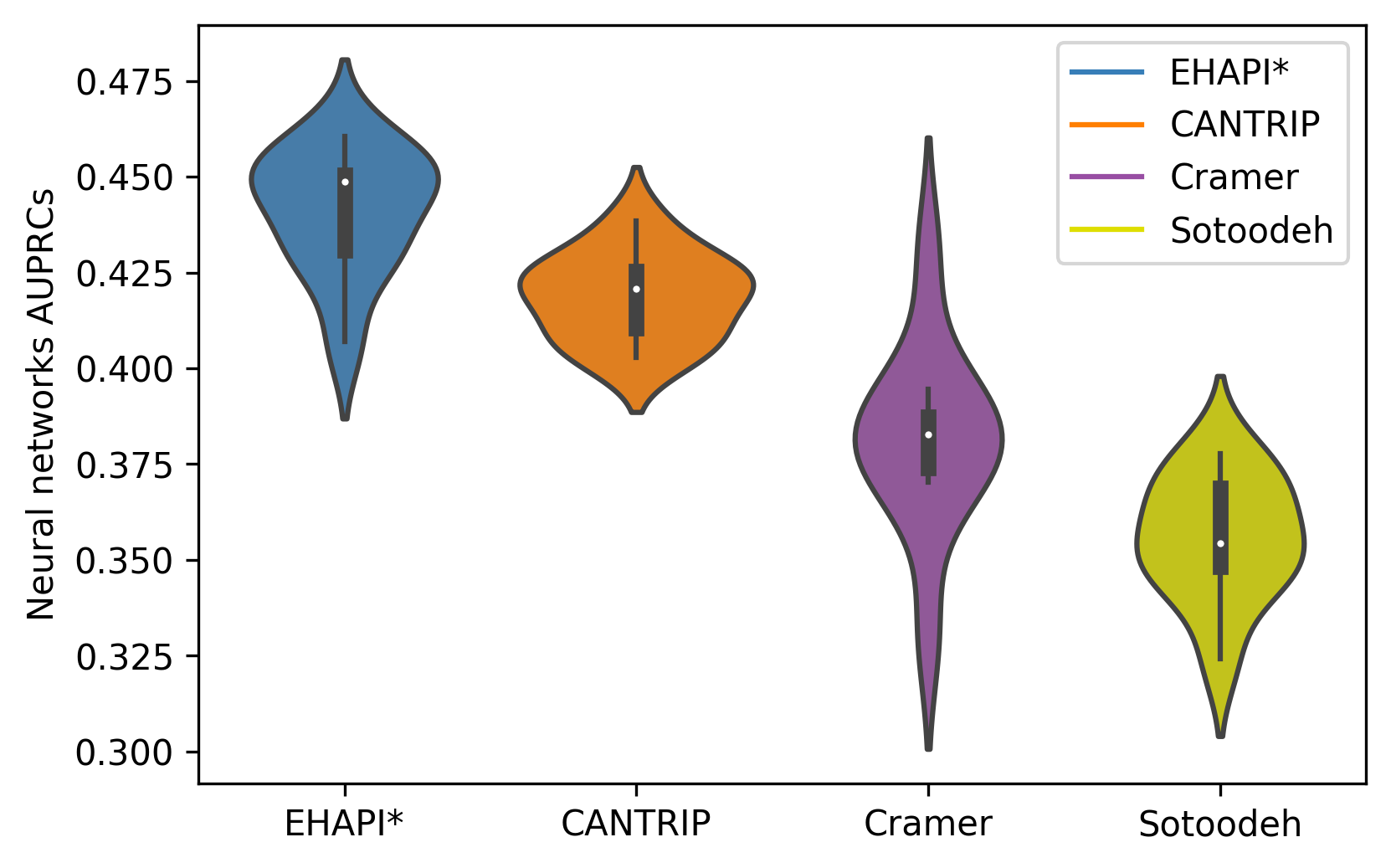
**

1. Neural network- AUPRC

**
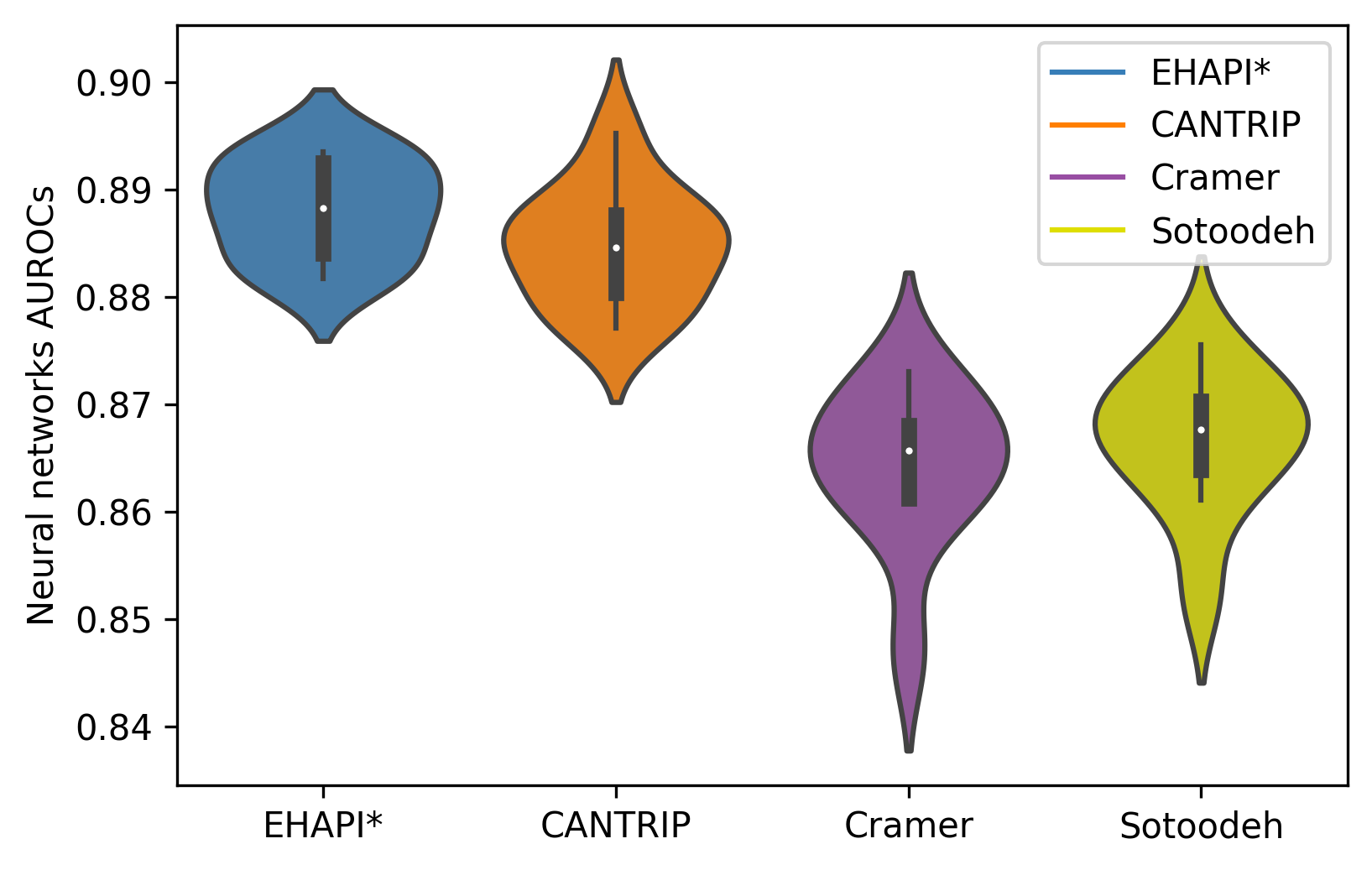
**

1. Neural network- AUROC

**Figure S6.** Test performance of definitions across 10 test sets and two metrics.

In Table S3, we compare the classification performance differences of the nurse labeled stays and the consensus stays for the 10 test sets. As can be seen from the results, EHAPI provides the best performance on the consensus datasets. However, Sotoodeh provides better results on the nursing labeled set compared with EHAPI. We note that for Sotoodeh, the standard deviation is higher and this suggests that the manually labeled set is too small to draw significant conclusions.

##

| *Definition* | *Label set* | *Gradient Boosting* | | *Neural Networks* | |
| --- | --- | --- | --- | --- | --- |
|  |  | *AUPRC* | *AUC* | *AUPR* | *AUC* |
| **EHAPI** | **Nurse** | 0.235*±*0.014 | 0.564*±*0.020 | 0.208*±*0.017 | 0.499*±*0.024 |
|  | **Consensus** | 0.507*±*0.019 | 0.911*±*0.004 | 0.464*±*0.015 | 0.899*±*0.006 |
| CANTRIP [10] | Nurse | 0.210*±*0.012 | 0.530*±*0.021 | 0.194*±*0.038 | 0.468*±*0.017 |
|  | Consensus | 0.495*±*0.023 | 0.907*±*0.004 | 0.464*±*0.015 | 0.899*±*0.005 |
| Cramer [6] | Nurse | 0.211*±*0.023 | 0.525*±*0.031 | 0.213*±*0.011 | 0.510*±*0.026 |
|  | Consensus | 0.380*±*0.023 | 0.887*±*0.003 | 0.412*±*0.037 | 0.880*±*0.010 |
| Sotoodeh [9] | Nurse | 0.270*±*0.049 | 0.606*±*0.049 | 0.244*±*0.035 | 0.561*±*0.044 |
|  | Consensus | 0.343*±*0.017 | 0.880*±*0.004 | 0.378*±*0.025 | 0.874*±*0.008 |

**Table S3.** Classifiers’ performance for the four HAPI definitions in MIMIC-III over 10 test sets broken down by the labeled set (nurse annotation or the consensus set).

## Section S11 Significant features for classifiers and definitions

For each definition, we analyzed useful words that were found to be most important by the two classifiers. For gradient boosting, we used the feature importance of the classifier directly. For the sequential neural network, the ‘shap’ [12] package was used with the first 1000 samples of the training data to explain the first positive test sample. Table S2 shows the non-general important features extracted. *EHAPI* definition identified the most specific HAPI terms among the definitions for both classifiers. The underline words can be clinically relevant and have sometimes been identified by multiple definition and classifier combinations.

| *Definition* | *Gradient boosting* | *Neural Networks* |
| --- | --- | --- |
| **EHAPI** | line, svc, vent, coarse, aspirin, suctioned, picc, lower, changes, disposition, coccyx, vanco, abgs, chamber, facility, tan, paralyzed, dialysis, osteomyelitis, wound, abscess, abnormalities | coherent, extended, motrin, qtc, syncope, bradycardia, plaque, fracture, pressure, valve, echo, ibuprofen, sat, reassess, ventricular, facility |
| CANTRIP [10] | doppler, line, picc, resp, lower, remains, ed, allergies, pa, tan, dictated, svc, weaning, tracing, coccyx, suctioned, residuals, abnormalities, breath, myocardial, fascicular | ambulatory, sutures, delayed, consciousness, instructions, asa, clopidogrel, facility, chlorhexidine, bisacodyl, shower, syncope, fracture, prolapse, disposition, plavix, sodium, injury, hgb, spinal, qtc, trauma, sat, bradycardia, stenosis |
| Cramer [6] | doppler, abg, paracentesis, habitus, peep, lower, anasarca, sedation, anesthesia, debridement, cavity, respiratory, weaning, coarse, paralyzed, desaturation, ciprofloxacin, fent | shower, sah, walking, clopidogrel, chlorhexidine, stairs, procedural, gluconate, aid, coumadin, hgb, consciousness, systolic, bisacodyl, central, perfusion, bid, veins, rehab, instructed, fluids, ultrasound, extremity, femoral, mid, syndrome |
| Sotoodeh [9] | duoderm, sacral, coccyx, wound, **picc,** consult, osteomyelitis, healing, diffuse, brachial, lower, rehab, pleural, pneumonia, subsegmental, nonionic, dressings, venous, fecal, addendum, tracing, abg, inability, redness, ams, sbt | infiltrate, prolonged, colace, stairs, superficial, consciousness, disposition, release, ambulatory, sah, sig, completed, ica, intake, contusion, carotid, stenosis, constipation, dvt, pulmonary, femoral, po, delayed, duplex |

### **Table S4.** Important words for each classifier by definition (sorted by importance)

***Glossary of terms used in Table S2***

**Svc** = Superior vena cava syndrome (SVCS) is obstruction of blood flow through the superior vena cava (SVC); symptoms include breathing problems, lightheadedness, and swelling in the upper body.

**Picc** = peripherally inserted central catheter.

**Vanco** =Vancomycin is used to treat colitis (inflammation of the intestine caused by certain bacteria) that may occur after antibiotic treatment.

**Abg** = arterial blood gases (ABG) test measures the acidity (pH) and the levels of oxygen and carbon dioxide in the blood from an artery

**Tan** =Tropical Ataxic Neuropathy

**Osteomyelitis** (OM) = an infection of bone; signs and symptoms of osteomyelitis include fever; swelling, warmth, and redness over the area of the infection; pain in the area of the infection, fatigue.

**Fascicular** = small bundle.

**Anasarca** = the skin and its underlying tissues retain salt and water, causing swelling all over the body.

**Habitus** = body build, and constitution especially as related to predisposition to disease.

**Peep** = Positive end-expiratory pressure (PEEP) is the positive pressure that will remain in the airways at the end of the respiratory cycle.

**Paracentesis** = A procedure in which a thin needle or tube is put into the abdomen to remove fluid from the peritoneal cavity.

**Fent** = fentanyl to treat severe pain.

**Ciprofloxacin** = antibiotic

**Sbt** = Spontaneous breathing trial (SBT) assesses the patient's ability to breathe while receiving minimal or no ventilator support.

**Ams** = Altered mental status (AMS)

**Brachial** = relating to, or situated in, the arm or an armlike process the brachial artery of the upper arm.

**Motrin** = trademark for ibuprofen.

**Qtc** = QT corrected for heart rate. The QT interval is the time from the beginning of the QRS complex, representing ventricular depolarization, to the end of the T wave, resulting from ventricular repolarization.

**Plavix** = Clopidogrel = Clopidogrel bisulfate = an anti-platelet drug.

**Hgb** = Hemoglobin (Hb or Hgb).

**Stenosis** = a narrowing or constriction of the diameter of a bodily passage or orifice.

**Sah** = Subarachnoid hemorrhage (SAH) is a life-threatening type of stroke caused by bleeding. into the space surrounding the brain.
**Ica** = internal carotid artery.

**Colace** = Colace (docusate) is a stool softener.

**Bisacodyl** = a mild laxative.
